# Gut Microbiota Composition in the First and Third Trimester of Pregnancy among Malay Women is Associated with Body Mass Index: A Pilot Study

**DOI:** 10.21315/mjms2023.30.1.10

**Published:** 2023-02-28

**Authors:** Bahiyah Abdullah, Mohd Yusri Idorus, Suzanna Daud, Shafiq Aazmi, Thanikasalam Kathiresan Pillai, Zaini Mohd Zain

**Affiliations:** 1 Maternofetal and Embryo (MatE) Research Group, Faculty of Medicine, Universiti Teknologi MARA, Sungai Buloh Campus, Selangor, Malaysia; 2 Department of Obstetrics and Gynaecology, Faculty of Medicine, Universiti Teknologi MARA, Sungai Buloh Campus, Selangor, Malaysia; 3 Institute of Medical Molecular Biotechnology, Faculty of Medicine, Universiti Teknologi MARA, Sungai Buloh Campus, Selangor, Malaysia; 4 School of Biology, Faculty of Applied Science, Universiti Teknologi MARA, Selangor, Malaysia; 5 Microbiome Health and Environment (MiHeaRT), Faculty of Applied Sciences, Universiti Teknologi MARA, Selangor, Malaysia; 6 Department of Medical Microbiology and Parasitology, Faculty of Medicine, Universiti Teknologi MARA, Selangor, Malaysia

**Keywords:** gut flora, taxonomic changes, microbial changes

## Abstract

**Background:**

This study has analysed the pattern of gut microbiota during the first and third trimesters among pregnant Malay women.

**Methods:**

This was a pilot prospective observational study involving 12 pregnant Malay women without any endocrine disorders and on neither antibiotics nor probiotics. Demographic details and anthropometric measurements were obtained, and the faecal 16S ribosomal ribonucleic acid (rRNA) metagenome microbiota of the first and third trimesters (T1 and T3) were analysed. Univariate and multivariate statistics, partial least squares discriminant analysis (PLSDA) and Kendall rank correlation testing were used to identify key genera and associations with pregnancy trimester and body mass index (BMI).

**Results:**

The most abundant phyla were Bacteroidetes, Firmicutes, Proteobacteria and Actinobacteria, with significant differences in composition at the genus level demonstrated between T1 and T3. Sequencing showed a statistically significant difference in beta diversity between normal and abnormal BMI at all taxonomic ranks (*R*^2^ = 0.60; *Q*^2^ = 0.23) and genus levels (*R*^2^ = 0.57; *Q*^2^ = 0.37). The relative abundances of Akkermansia (*P* < 0.05; false discovery rate [FDR] < 0.05), Olsenella (*P* < 0.05; FDR < 0.05) and Oscillospira (*P* < 0.05; FDR < 0.05) were found to be significantly higher in normal BMI cases by 2.4, 3.4 and 3.1 times, respectively.

**Conclusion:**

Three genera (Akkermansia, Olsenella and Oscillospira) were correlated with normal BMI during pregnancy. All three could be promising biotherapeutic targets in body weight regulation during pregnancy, subsequently reducing complications associated with higher BMI.

## Introduction

The gut is colonised by millions, billions or even trillions of bacteria, most of them commensals. The exact composition of gut microbiota is unknown, but recent investigations have shown that 80%–90% of bacteria phenotypes are members of two phyla: the Bacteroides and the Firmicutes (1). Studies have shown that, in addition to dietary intake, antibiotics, stress and obesity, the gut flora may also be altered by pregnancy (1).

Pregnancy is a remarkable biological process involving simultaneous changes in many physiological systems. While some of the metabolic, hormonal changes have been known for decades, the dramatic change in gut microbiota during pregnancy has only recently been appreciated (2). Koren et al. (3) have reported an increase in bacterial load and significant changes in the gut microbiota’s composition, particularly in late gestation. In their study, individual richness (α-diversity) decreased, between-subject diversity (β-diversity) increased and the abundance of particular species changed dramatically. These factors were coupled with weight gain, insulin insensitivity and a higher level of cytokines reflecting inflammation. The authors postulated that these changes, which resembled the changes seen in metabolic syndrome, were a necessary functional change to accommodate normal pregnancy demand (3). In another study, Neuman and Koren (4) found that gut microbiota may have played a role in maternal weight gain by enhanced glucose and fatty acid absorption, increased fasting-induced adipocyte factor release, the activation of catabolic pathways and immune system stimulation.

Maternal gut microbiota has also been reported to significantly affect infants’ immune systems; Neuman and Koren (4) hypothesised that knowledge about maternal gut microbiota during pregnancy is thus crucial in preventing allergic disease among newborns.

Few studies have reported on the taxonomic patterns of the gut microbiota for both the first and third trimesters (T1 and T3), although one study found that different ethnicities and dietary cultures were associated with varying microbiota compositions (5, 6). To date, research is lacking on a number of ethnic groups, including Malay women. This study has thus been executed to determine the pattern of gut flora during T1 and T3 among pregnant Malay women.

## Methods

### Study Design

For this pilot prospective observational study, we recruited 12 Malay women from the Gut Flora Study at Universiti Teknologi MARA Medical Centre and Sungai Buloh Hospital. The inclusion criteria were: i) pregnant Malay patients in T1 and ii) willingness to be followed up until the T3. The exclusion criteria were if a woman: i) had any known case of diabetes, metabolic syndrome or any other endocrine disorders or ii) had been on any antibiotics or probiotics within 4 weeks prior to recruitment.

The participants’ basic demographic details and anthropometric measurements were collected using a designated study pro forma. Participants were asked to provide stool samples during T1. Sample collection, preservation and storage were performed using stool nucleic acid collection and preservation tubes (Norgen, Canada). A total of 2 g of samples were collected and added to the collection tubes. Next, the stool was gently mixed until it was well submerged under the liquid preservative. Later, in T3, the participants were asked to provide another stool sample using the same kit.

### DNA Extraction

The total DNA of the stool samples was extracted from approximately 400 μL of liquid samples using the Stool DNA Isolation Kit (Norgen, Canada) following the manufacturer’s instructions. Final DNA concentration and purity were determined by a SpectraMax QuickDrop Micro-Volume Spectrophotometer (Molecular Devices, USA). The ratio of sample absorbance at 260 nm and 280 nm was used to assess the purity of the DNA. The DNA integrity was assessed by running 1% agarose gel electrophoresis (Sigma-Aldrich, USA) and then stained with SYBR Safe DNA Gel Stain (Invitrogen, USA). The extracted DNA was stored at −20 °C pending sequencing analysis.

### Two-Step Tailed Amplification Strategy

The V3–V4 hypervariable regions of the bacteria 16S ribosomal ribonucleic acid (rRNA) metagenome gene were amplified with a set of primers (338F [5′-ACTCCTACGGGAGGCAGCA-3′] and 806R [5′-GGACTACHVGGGTWTCTAAT-3′]) by a thermocycler polymerase chain reaction (PCR) system, with 27 cycles for each sample (GeneAmp 9700, ABI, USA). The PCR reactions were conducted using the following conditions: 3 min of denaturation at 95 °C, 27 cycles of 30 s at 95 °C, 30 s for annealing at 55 °C, 45 s for elongation at 72 °C and a final extension at 72 °C for 10 min.

The PCR amplification was performed using TransStart FastPfu DNA polymerase (TransGen AP221-02) under a 20 μL reaction containing 4 μL of 5 × FastPfu buffer, 2 μL of 2.5 mM dNTPs, 0.8 μL of each primer (5 μM), 0.4 μL of FastPfu polymerase, 0.2 μL bovine serum albumin and 10 ng of template DNA. The PCR products were detected by gel electrophoresis in 2% agarose gel. Amplicons were extracted from the agarose gels and purified using the AxyPrep DNA Gel Extraction Kit (Axygen Biosciences, USA) and quantified using QuantiFluor-ST (Promega, USA) following manufacturer protocols.

### 16S rRNA Metagenome Analysis

The sample library was pooled in equimolar and paired-end sequenced (2 × 300) on an Illumina MiSeq platform (Illumina, USA) according to the standard protocols by Majorbio Bio-Pharm Technology Co. Ltd. (Shanghai, China). Assembly, binning and annotation of DNA sequences were then performed. The raw FASTQ files were demultiplexed and quality-filtered using QIIME (version 1.9.1) with the following criteria: The 300 bp reads were truncated at any site that received an average quality score of < 20 over a 50 bp sliding window, discarding any truncated reads that were shorter than 50 bp; exact barcode matching was enforced, as was two-nucleotide mismatch in primer matching; reads containing ambiguous characters were removed; only sequences that overlapped longer than 10 bp were assembled according to their overlap sequence; reads that could not be assembled were discarded.

The taxonomy of each 16S rRNA gene sequence was analysed by RDP Classifier against the Silva (SSU123) 16S rRNA database using a confidence threshold of 0.7. A species accumulation curve at the operational taxonomic unit (OTU) level was used to assess the sequencing depth and species richness from the results of the sampling. Several alpha diversity indices, including Chao 1 richness, the abundance-based coverage estimator (ACE) metric, the Shannon-Wiener curve and the Simpson index, were calculated using mothur.

### Statistical and Comparative Metagenomics Analysis

Clinical baseline characteristics are presented as the mean, with standard deviation (SD) and median within the mean’s range. Statistical analysis was performed using SPSS software, version 22 (SPSS, Chicago, IL, USA). Due to the small sample size, the distribution of the data was evaluated using Shapiro-Wilk testing; a *P*-value greater than 0.05 was determined to be normally distributed data. Differences in alpha diversity indices (Chao 1 richness, ACE metric, Shannon-Wiener curve and Simpson index) between T1 and T3 were evaluated using paired-sample *t*-testing. Statistical significance was defined as a *P*-value of < 0.05.

Comparative metagenomics analyses between the 16S rRNA gut microbiota profiles of T1 and T3 were performed using METAGENassist (7). Unassigned and unmapped reads and low-count OTUs with less than 10% occurrences and with more than 50% missing reads were filtered out. Row-wise normalisation by sum was performed on the bacterial relative abundance data matrix to normalise the inherent differences within metagenomes (sequencing depth). Column-wise normalisation by log_10_ transformation was employed to obtain a more normal/Gaussian distribution of each of the bacterial taxa (8).

A supervised model partial least squares discriminant analysis (PLSDA) was used to reveal microbiota variation and to identify the key genera that were responsible for different microbiota structures between pregnancy trimesters (T1 versus T3) and body mass index (BMI) groups (normal versus abnormal). The discriminant patterns from the PLSDA model were ascertained by *R*^2^ and *Q*^2^ values. The importance of phyla and genera in the clustering pattern of microbiota structure between pregnancy trimesters and BMI groups was determined using variable importance in projection (VIP). For VIP, variables with values of 1.5 and above were considered to be important contributors to the observed discriminant patterns.

Differences in relative abundances of gut microbiota at the phylum and genus levels between pregnancy trimesters and BMI groups were analysed using univariate statistics such as fold change analysis (threshold set at 2.0), paired-samples *t*-testing and independent sample *t*-testing with significance values of *P* < 0.05. To correct for false positives, a false discovery rate (FDR) of < 0.05 was set as the significance threshold. Kendall rank correlation testing was used to assess associations between the key genera-level relative abundances identified from the PLSDA model and patient characteristics.

## Results

### Characteristics of the Participants

Twelve participants were included in this pilot study. Table 1 shows the participants’ demographics. Those who were overweight, pre-obese or obese were categorised as having abnormal BMI for this study.

### Gut Microbiota Structure at T1 and T3

A total of 1,610,705 high-quality sequences with an average sequence length of 438 bp were generated from 24 stool samples clustered to OTUs at 97% sequence identity. A total of 587 OTUs were obtained from the 1,610,705 high-quality sequences. The species accumulation assessment using the Shannon-Wiener curve showed a plateau and a saturation phase, indicating sufficient sequencing depth, and the sample size was sufficient to capture the overall richness of the gut microbiota structure examined in this study. No significant differences were noted in alpha diversity richness from Chao 1 (*P* = 0.630), the ACE metric (*P* = 0.619), Shannon-Wiener curve (*P* = 0.784) or the Simpson index (*P* = 0.484) between T1 and T3 (Table 2).

The most abundant phyla observed during T1 were Bacteroidetes (47%), followed by Firmicutes (43%), Proteobacteria (7%) and Actinobacteria (2%). During T3, the most abundant phyla were Firmicutes (45%), followed by Bacteroidetes (43%), Proteobacteria (8%) and Actinobacteria (1%). These prevalent phyla represented 99% and 97% of gut microbiota composition in T1 and T3, respectively. Comparative analysis of microbial taxa abundance at the phylum level showed an increase of Firmicutes by 2% and a reduction of Bacteroidetes by 4% during T3 as compared to T1. No significant difference was noted in gut microbiota composition at the phyla level between T1 and T3, however.

### Distinct Composition of the Gut Microbiota during T1 and T3

**Figure 1:**
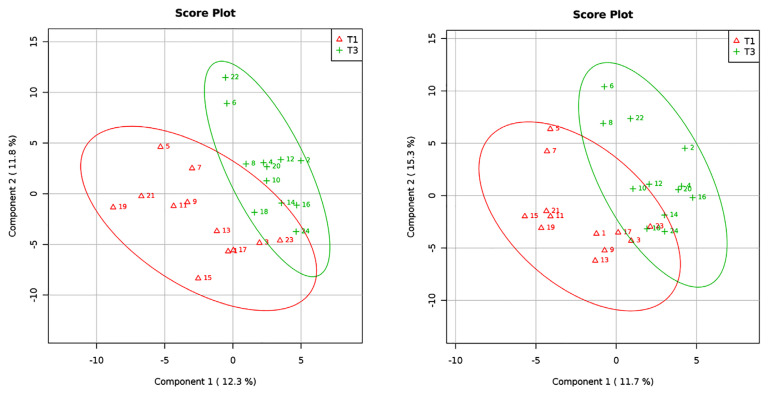
Partial Least Square Discriminant Analysis (PLSDA) shows a clustering pattern of gut microbiota according to first-trimester pregnancy (T1) and third-trimester pregnancy (T3) at all taxonomical rank (A) and at the genus level (B). Each point represents a sample and points with the same colour belong to the same group. The red points represent T1 and the green points represent T3. The line ellipses indicate the 95% confidence interval

**Figure 2:**
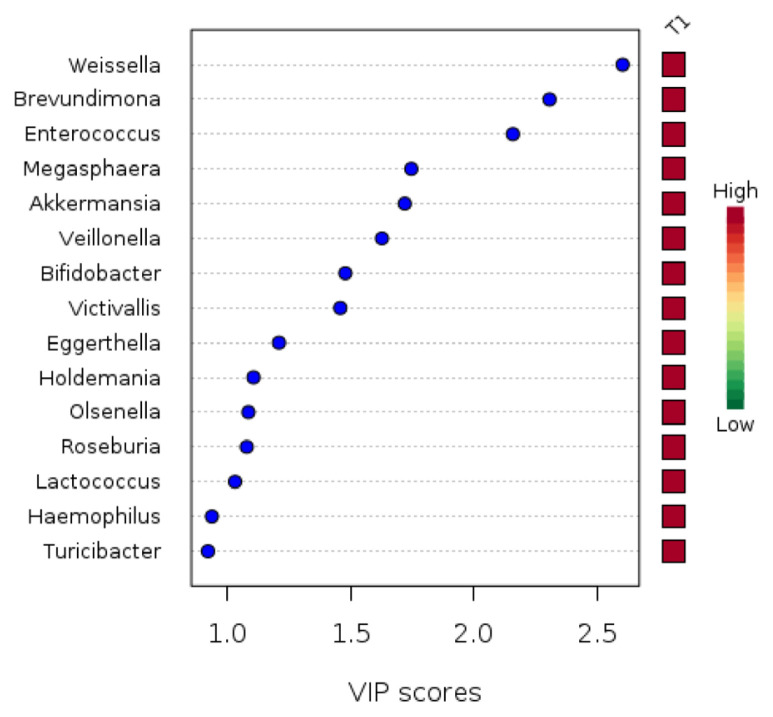
The key genera for clustering of gut microbiota according to pregnancy trimester with variable importance in projection (VIP) score more than 1.5. The scores were calculated based on the top five components determined by cross-validation. The coloured boxes on the right indicate the relative abundances of the corresponding taxa in each group under study

Using a squares regression model, PLSDA showed compositional differences of gut microbiota at all taxonomic ranks (*R*^2^ = 0.46; *Q*^2^ = −0.89) and at the genus level (*R*^2^ = 0.53; *Q*^2^ = −0.48) between T1 and T3 (Figure 1). The abundances of 44 genera were found to be responsible for the clear clustering pattern of gut microbiota according to the pregnancy trimester observed in the PLSDA model (Supplementary Table 1). Of the 44 genera, only eight (*Weissella*, *Brevundimonas*, *Enterococcus*, *Megasphaera*, *Akkermansia*, *Veillonella*, *Bifidobacterium* and *Victivallis*) had VIP scores of 1.5 and above (Figure 2). Fold change analysis revealed that the abundance of *Brevundimonas* was 2.4 times higher in T1 compared to T3. The abundances of these eight key genera were not found to be statistically significantly different between T1 and T3, however.

### Gut Microbiota Composition during T1 and T3 were Correlated with BMI

At the phylum level, the relative abundance of Bacteroidetes was higher among participants with normal BMI (48.9%) than with abnormal BMI (44.7%). No difference was noted in relative abundances between normal and abnormal BMI for Firmicutes, however. Interestingly, the relative abundance for the Proteobacteria phylum was higher among participants with abnormal BMI (7.1%) than those with normal BMI (5.3%).

**Figure 3:**
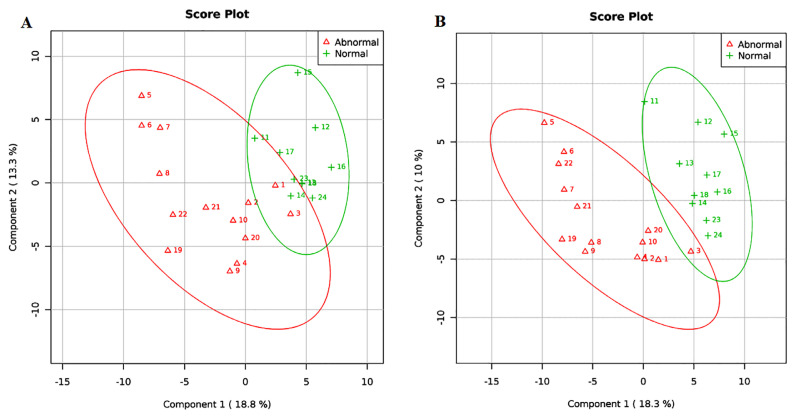
Clustering of gut microbiota composition on Partial Least Square Discriminant Analysis (PLSDA) model according to body mass index (BMI) of the participants at all taxonomical rank (A) and at the genus level (B). Each point represents a sample and points with the same colour belong to the same group. The green points represent normal BMI (18.5 kg/m2–22.9 kg/m2) and red points represent abnormal BMI (overweight: 23 kg/m2–24.9 kg/m2; pre-obese: 25.0 kg/m2–29.9 kg/m2; obese: > 30 kg/m2). The line ellipses indicate the 95% confidence interval

Using a squares regression model, PLSDA showed compositional differences of gut microbiota at all taxonomic ranks (*R*^2^ = 0.60; *Q*^2^ = 0.23) and genus levels (*R*^2^ = 0.57; *Q*^2^ = 0.37) between participants from the normal and abnormal BMI groups (Figure 3). A total of 44 genera were found to be important variables in discriminating the gut microbiota structure between abnormal and normal BMI during pregnancy (Supplementary Table 2).

**Figure 4:**
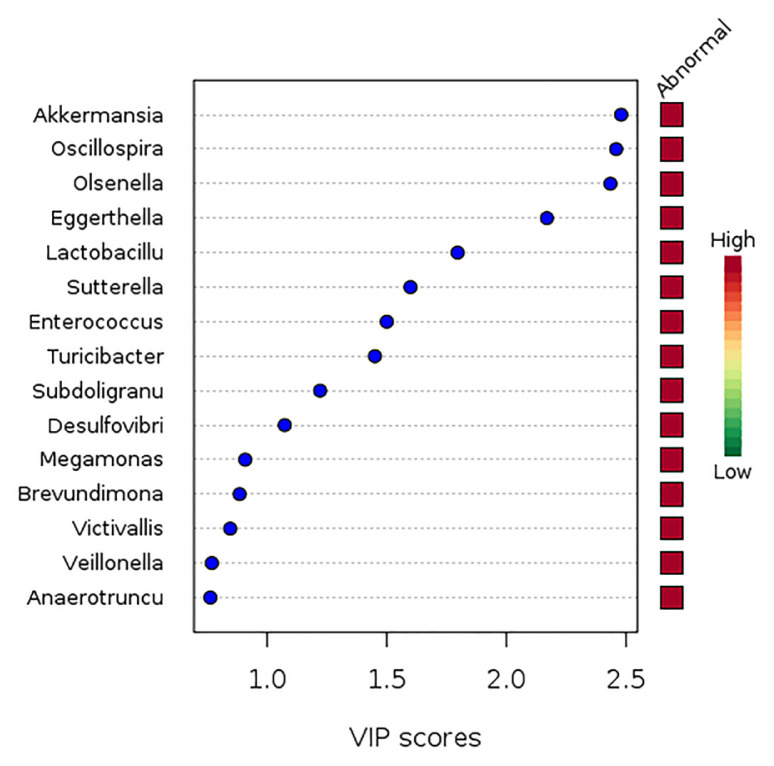
The key genera for clustering of gut microbiota according to BMI of the participants with variable importance in projection (VIP) score more than 1.5. The score was calculated based on the top five components determined by crossvalidation. The coloured boxes on the right indicate the relative abundances of the corresponding taxa in each group under study

**Figure 5:**
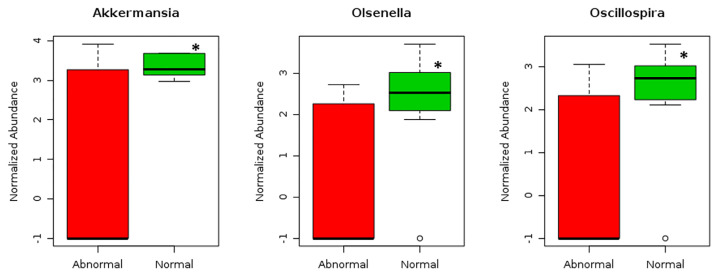
The normalised abundance of Akkermansia, Olsenella and Oscillaspira are significantly higher in the normal BMI group as compared to the abnormal BMI group during pregnancy. *Independent samples *t*-test (*P* < 0.05, FDR = 0.05)

Only eight genera with VIP scores of 1.5 and above were identified as key genera in the gut microbiota structure shown in Figure 3, however. The eight key genera were *Akkermansia*, *Oscillospira*, *Olsenella*, *Eggerthella*, *Lactobacillus*, *Sutterella*, *Enterococcus* and *Turicibacter* (Figure 4). Of the eight key genera, only the abundance of *Akkermansia* (*P* < 0.05; FDR < 0.05), *Olsenella* (*P* < 0.05; FDR < 0.05) and *Oscillospira* (*P* < 0.05; FDR < 0.05) were found to be significantly higher among the normal BMI group compared to the abnormal BMI group, by 2.4, 3.4 and 3.1 times, respectively, based on fold change analysis (Figure 5).

Correlation between the identified key genera-level relative abundances and BMI was further investigated. Interestingly, a negative correlation was found between BMI and *Akkermansia* (*P* < 0.05; tau = −0.413; Kendall rank correlation testing), *Olsenella* (*P* < 0.05; tau = −0.601; Kendall rank correlation testing) and *Oscillospira* (*P* < 0.05; tau = −0.593; Kendall rank correlation testing).

## Discussion

This pilot study found that the most abundant phylum observed during T1 was Bacteroidetes (46%), followed by Firmicutes (43%), Proteobacteria (7%) and Actinobacteria (2%). Previous studies have demonstrated that the gut microbiota composition during T1 is similar to that of the non-pregnant population. Bacteroidetes and Firmicutes have been demonstrated to be two major components. In this study, there was no significant difference in the microbiota composition between T1 and T3.

The most abundant phylum in T3 was Firmicutes (45%), followed by Bacteroidetes (43%), Proteobacteria (8%) and Actinobacteria (1%). Koren et al. (3) previously showed an increased abundance of the Actinobacteria and Proteobacteria phyla in the third trimester. The present study did find a similar rising trend of Proteobacteria but a decreasing trend of Actinobacteria. The increased abundance of Proteobacteria that we observed in T3 has been repeatedly demonstrated to be associated with inflammation-associated dysbiosis (9), thus suggesting the existence of underlying inflammation as part of physiological adaptation during T3.

Similar to our findings, DiGiulio et al. (10), who collected rectal swabs weekly from the end of T1 to delivery among 40 women in the USA, also did not demonstrate any significant changes in the relative abundance of gut microbiota taxa. Another study involving 56 Tanzanian women in which monthly stools were collected from 12 weeks–24 weeks of pregnancy to delivery also found no significant differences in the relative abundance of specific taxa of the gut microbiota (11).

Further analysis of our study results demonstrated compositional differences of gut microbiota at the genus level between T1 and T3. Eight genera were identified as the most important contributors to the clustering of gut microbiota composition: *Weissella*, *Brevundimonas*, *Enterococcus*, *Megasphaera*, *Akkermansia*, *Veillonella*, *Bifidobacterium* and *Victivallis*. Interestingly, our study showed that the abundance of *Brevundimonas* in first trimester was 2.4 times higher than in second trimester. *Brevundominas* is a gram-negative, non-fermenting, aerobic bacteria. No previous studies have been conducted on its role in pregnancy. Most available literature has demonstrated *Brevundimonas* as being pathogenic in many clinical situations (12), although its presence and increased patterns in the gut during pregnancy still lack an explanation.

This study has also demonstrated that there was no significant difference in alpha diversity by observation using Chao 1 richness, the ACE metric, the Shannon-Wiener curve and the Simpson index, although the trend was toward reduced species richness. Koren et al. (3) demonstrated a significant reduction in alpha diversity, while DiGiulio et al. (10) observed only marginal decreases in alpha diversity. Individuals with low species richness were associated with more marked overall adiposity, insulin resistance and dyslipidaemia and a more pronounced inflammatory phenotype when compared with those with high species richness (13). Despite appearing to be non-beneficial to the host’s health, these functional changes could be a necessity to accommodate increased energy demand during T3. This finding, however, leads to concerns about whether exaggeration of these functional changes could increase the risk of gestational diabetes.

Maternal gut microbiota has also been known to be associated with maternal BMI (14–17). Maternal obesity during pregnancy has been associated with alterations in the composition and diversity of the intestinal microbial community. The present study has demonstrated that pregnant women with abnormal BMI (overweight, pre-obese or obese) were associated with a lower abundance of Bacteroidetes and a higher abundance of Proteobacteria at the phylum level. No significant difference in Firmicutes was noted. Our findings contradict those of Gomez-Arango et al. (18), who compared the microbial composition between obese and overweight women; they found significantly higher relative abundances of Firmicutes and Actinobacteria and lower relative abundances of Tenericutes. Interestingly, the pattern of taxonomic changes noted in the present study was different, which could have been due to the different ethnicities examined between the two studies.

We did note compositional differences of gut microbiota at the genus level between normal and abnormal BMI pregnant women in our study. Although our sample size is small, the reliability of the PLSDA model (Figure 3) on the discriminant pattern of gut microbiota observed between normal and abnormal BMI during pregnancy was ascertained based on the value of *R*^2^ and *Q*^2^, both of which showed positive values. In contrast, the reliability of the PLSDA model of gut microbiota structure according to pregnancy trimester (Figure 1) could not be ascertained, as *Q*^2^ was negative in value. This finding indicates a lower prediction relevance of the mathematical model in discriminating the pattern of gut microbiota based on pregnancy trimesters.

We identified eight key genera (*Akkermansia*, *Oscillospira*, *Olsenella*, *Eggerthella*, *Lactobacillus*, *Sutterella*, *Enterococcus* and *Turicibacter*) that contributed to the clustering of gut microbiota composition based on BMI grouping. A detailed analysis found that *Akkermansia*, *Olsenella* and *Oscillospira* were statistically significantly higher by factors of 2.4, 3.4 and 3.1, respectively, among normal BMI pregnant women than the abnormal BMI group. Because these three key genera all showed a negative correlation with BMI, it is tempting to speculate that these gut bacteria mediate body weight regulation during pregnancy.

*Akkermansia* sp. has been inversely associated with obesity, diabetes, inflammation and metabolic disorders (19–21). The gut bacteria *Akkermansia* has been identified as a promising probiotic due to its reported antiobesity effects among human and animal studies through the regulation of lipid metabolism, immune response and gut systems (19–21). The increment of *Akkermansia* among the normal BMI pregnant women in our study may support the potential causal benefits of body weight regulation during pregnancy.

The gut bacteria *Olsenella* from the phylum Firmicutes has just recently been discovered from the human gut and has been identified as one of the butyrate-producing bacteria (22, 23). Butyrate is one of the small organic short-chain fatty acids (SCFAs) produced via the fermentation of dietary fibres by gut bacteria such as *Olsenella* (23, 24). The reported health benefits of butyrate are that it provides fuel for the gut epithelial cells, mediates the immune system functions of the colon wall and protects against certain diseases of the digestive tract such as inflammatory bowel disease (24, 25). Aberration in the production of butyrate has also been correlated with obesity and metabolic diseases; this finding has emerged as a promising development in biotherapeutics (24).

*Oscillospira* is among the gut mucosa–associated bacteria (25) to show a negative correlation with BMI (26, 27) and a positive correlation with leanness (28). Haro et al. (29), however, have reported contradictory findings on the higher abundance of *Oscillospira* among obese people following a Mediterranean diet. The distinct composition of *Akkermansia*, *Olsenella* and *Oscillospira* reported in the present study should be investigated further for their biotherapeutic potential on obesity.

This is the first study to have been performed among a Malay population from Southeast Asia; a comparison between similar populations thus could not be performed. Dwiyanto et al. (6) have demonstrated that ethnicity independently influenced the gut microbiota among infants with a higher abundance of lactic acid bacteria among South Asians and a higher abundance of genera within the order Clostridiales among white Caucasians. The same study also showed that infant-feeding practices also significantly influenced the gut microbiota, meaning that the different dietary practices of Malay women could also have been a contributing factor.

Our study does have certain limitations. Gut microbiota composition is influenced by diet. Dietary information through a food-frequency questionnaire was not obtained in this study, so any relationships between dietary intake and microbiota composition could not be examined. Our exclusion of women who were on antibiotics or probiotics was a strength of the study, however, in light of previous studies that have demonstrated that antibiotic use may alter the gut microbiota composition and reduce its diversity (30, 31). Our pilot study used a small sample size, so the results may not represent the whole population of pregnant Malay women, although the current findings will be beneficial for illuminating gut microbiota compositions and patterns, particularly among the population of pregnant Malay women.

## Conclusion

Compositional differences of gut microbiota were noted at the genus level between the T1 and T3 of pregnancy, with a decreasing pattern of alpha diversity also noted. We also noted compositional differences of gut microbiota at the genus level between women in different BMI groups; *Akkermansia*, *Olsenella* and *Oscillospira* were found to be negatively correlated with BMI. These key genera could thus be promising new-generation probiotics in the context of body weight regulation during pregnancy.

## Supplementary Information

**Supplementary Table 1:** 

Genus	Comp. 1	Comp. 2	Comp. 3	Comp. 4	Comp. 5
Weissella	2.6032	2.2506	2.1174	2.0418	1.9897
Brevundimonas	2.3064	1.9997	1.898	1.8413	1.8231
Enterococcus	2.1577	1.9865	1.9105	1.8436	1.7972
Megasphaera	1.7452	1.5581	1.4743	1.4846	1.4508
Akkermansia	1.7188	1.7838	1.7157	1.6678	1.63
Veillonella	1.626	1.3958	1.3335	1.295	1.2788
Bifidobacterium	1.4775	1.2734	1.3888	1.3389	1.3052
Victivallis	1.4569	1.3597	1.2844	1.2801	1.2848
Eggerthella	1.2079	1.6205	1.5463	1.4972	1.4602
Holdemania	1.1046	0.99031	0.93783	1.0303	1.1446
Olsenella	1.0839	1.4598	1.4362	1.3905	1.3568
Roseburia	1.0771	1.0205	1.0759	1.0565	1.0527
Lactococcus	1.0296	0.99377	0.93536	0.91468	1.1037
Haemophilus	0.9356	0.8214	0.86148	0.8889	0.89167
Turicibacter	0.9196	0.78236	0.7425	0.9853	0.98428
Clostridium	0.85657	0.76722	0.79082	1.0917	1.0686
Oscillospira	0.84379	1.3657	1.3377	1.2998	1.2679
Lactobacillus	0.69364	0.58785	1.0004	0.97319	1.0151
Faecalibacterium	0.68647	0.60542	0.57713	0.58763	0.57321
Anaerotruncus	0.61896	0.56899	0.55418	0.53529	0.52396
Acidaminococcus	0.59623	0.95767	0.92917	0.89575	1.0457
Collinsella	0.59606	0.52033	0.49389	0.49162	0.56415
Dorea	0.59551	0.5064	0.56984	0.54962	0.53566
Alistipes	0.57532	0.58073	0.61683	0.62037	0.62654
Phascolarctobacterium	0.55406	0.6838	0.64946	0.62872	0.66022
Megamonas	0.54012	0.48359	0.68775	0.69822	0.72386
Butyricicoccus	0.51702	0.4533	0.43477	0.42792	0.41937
Anaerostipes	0.48793	0.55112	0.52331	0.52624	0.61204
Citrobacter	0.47454	0.42658	0.44277	0.43291	0.42187
Barnesiella	0.46813	0.82971	0.78106	0.77952	0.80815
Achromobacter	0.46381	0.72262	0.71071	0.81163	0.79193
Butyricimonas	0.44239	0.54339	0.51307	0.78676	0.76676
Prevotella	0.38442	0.37526	0.56067	0.54383	0.53002
Subdoligranulum	0.26028	0.28888	0.68934	0.6751	0.68471
Blautia	0.18167	0.21075	0.2006	0.19636	0.19163
Peptostreptococcus	0.17014	0.14674	0.69458	0.68555	0.66915
Sutterella	0.16043	0.8673	1.0137	0.98531	0.96025
Bacteroides	0.13351	0.12532	0.12502	0.13354	0.14596
Dialister	0.13257	0.88262	0.90557	0.94108	0.95786
Parabacteroides	0.1278	0.1251	0.1844	0.18602	0.18212
Odoribacter	0.10041	0.32889	0.47203	0.51998	0.54246
Desulfovibrio	0.039951	0.85608	0.92498	0.95467	0.93252
Lachnospira	0.017792	0.16611	0.15787	0.15434	0.15551
Streptococcus	0.011874	0.1304	0.13155	0.12794	0.12517

**Supplementary Table 2:** 

Genus	Comp. 1	Comp. 2	Comp. 3	Comp. 4	Comp. 5
Akkermansia	2.478	2.2191	2.0417	1.9902	1.9643
Oscillospira	2.4572	2.201	2.121	2.0868	2.0679
Olsenella	2.4334	2.157	1.9876	1.9504	1.9351
Eggerthella	2.1681	1.9276	1.9069	1.8635	1.8399
Lactobacillus	1.7951	1.5951	1.532	1.5023	1.5083
Sutterella	1.5984	1.491	1.4713	1.4316	1.4441
Enterococcus	1.4996	1.3314	1.2173	1.2102	1.1957
Turicibacter	1.4499	1.3999	1.3162	1.2816	1.2652
Subdoligranulum	1.221	1.1446	1.0693	1.0554	1.0671
Desulfovibrio	1.0734	1.2484	1.1434	1.1512	1.1465
Megamonas	0.90855	1.0772	1.0216	0.99237	0.9794
Brevundimonas	0.88504	0.78671	0.87565	0.89654	0.8882
Victivallis	0.84588	1.0289	0.97751	1.0456	1.043
Veillonella	0.76905	0.81586	0.77779	0.76027	0.7512
Anaerotruncus	0.763	0.69543	0.64604	0.63862	0.6303
Odoribacter	0.75798	0.69808	0.67423	0.6575	0.65593
Anaerostipes	0.75693	0.67643	0.65517	0.63667	0.65882
Prevotella	0.75429	0.76792	0.72151	0.75542	0.79605
Alistipes	0.73381	0.75827	0.69342	0.69819	0.69361
Lactococcus	0.7116	0.63087	0.63182	0.75586	0.74885
Haemophilus	0.664	1.0612	1.0116	0.98252	1.007
Collinsella	0.6391	0.67976	0.69661	0.70513	0.71778
Barnesiella	0.4835	0.72228	0.6785	0.67942	0.76157
Holdemania	0.40591	0.71404	1.0901	1.1518	1.1583
Megasphaera	0.3736	1.4549	1.3521	1.3384	1.3241
Clostridium	0.32894	0.29313	0.5322	0.75668	0.77269
Blautia	0.31653	0.28341	0.26638	0.25879	0.25623
Weissella	0.30502	0.39373	0.64898	0.64125	0.6823
Acidaminococcus	0.30351	0.6813	1.3603	1.326	1.314
Bacteroides	0.26243	0.23371	0.21495	0.21369	0.21232
Dialister	0.24466	0.73261	0.84056	0.90969	0.9132
Achromobacter	0.23318	1.1177	1.0354	1.0185	1.0101
Peptostreptococcus	0.18951	0.29783	0.75081	0.79568	0.78647
Lachnospira	0.1784	0.17297	0.18187	0.1775	0.17775
Faecalibacterium	0.17365	0.30841	0.31178	0.36302	0.36845
Butyricimonas	0.17204	0.25596	0.54345	0.65307	0.7385
Streptococcus	0.14542	0.17507	0.16168	0.15807	0.15736
Parabacteroides	0.11342	0.10954	0.14757	0.16612	0.16772
Roseburia	0.11154	0.19129	0.31027	0.37963	0.3786
Bifidobacterium	0.10297	0.30627	0.42416	0.51748	0.54309
Butyricicoccus	0.0824	0.11835	0.11443	0.19831	0.23595
Dorea	0.074841	0.10076	0.63619	0.63279	0.62451
Phascolarctobacterium	0.070302	0.39682	0.3682	0.35859	0.35757
Citrobacter	0.062746	0.061418	0.43956	0.45006	0.44685

## Figures and Tables

**Table 1:** Participant demographics (*n* = 12)

Variables	*n* (%)
Age (years)^a^	32.8 (3.8)
Gravida^b^	1.5 (1–5)
Parity^b^	0.5 (0–3)
BMI (kg/m^2^)^a^	25.4 (5.9)
Normal	5 (41.7)
Overweight	1 (8.3)
Pre-obese	4 (33.3)
Obese	2 (16.7)
Blood pressure (BP) (mmHg)	
Systolic BP^a^	114.5 (13.3)
Diastolic BP^a^	68.9 (6.9)

Notes:

a mean (SD);

b median (range)

**Table 2:** Alpha diversity of the gut microbiota in first pregnancy trimester (T1) and third pregnancy trimester (T3)

Variable	Mean (SD) *n* = 12		Mean difference (95% CI)	*t*-statistic (df)	*P*-value*

	T1	T3			
Chao1	237.6 (76.28)	245.7 (70.97)	−8.1 (−44.3, 28.0)	−0.50 (11)	0.630
ACE	231.3 (73.58)	244.6 (68.71)	−8.3 (−44.2, 27.5)	−0.51 (11)	0.619
Shannon	3.2 (0.53)	3.2 (0.61)	0.1 (−0.4, 0.6)	0.28 (11)	0.784
Simpson	0.1 (0.05)	0.1 (0.10)	−0.0 (−0.1, 0.1)	−0.72 (11)	0.484

Notes: SD = standard deviation;

* paired-sample *t*-test

## References

[1] Cani PD, Delzenne NM (2009). Interplay between obesity and associated metabolic disorders: new insights into the gut microbiota. Curr Opin Pharmacol.

[2] Nuriel-Ohayon M, Neuman H, Koren O (2016). Microbial changes during pregnancy, birth, and infancy. Front Microbiol.

[3] Koren O, Goodrich Julia K, Cullender Tyler C, Spor A, Laitinen K, Kling Bäckhed H (2012). Host remodeling of the gut microbiome and metabolic changes during pregnancy. Cell.

[4] Neuman H, Koren O (2017). The pregnancy microbiome. Nestle Nutr Inst Workshop Ser.

[5] Stearns JC, Zulyniak MA, de Souza RJ, Campbell NC, Fontes M, Shaikh M (2017). Ethnic and diet-related differences in the healthy infant microbiome. Genome Med.

[6] Dwiyanto J, Ayub Q, Lee SM, Foo SC, Chong CW, Rahman S (2021). Geographical separation and ethnic origin influence the human gut microbial composition: a meta-analysis from a Malaysian perspective. Microb Genom.

[7] Arndt D, Xia J, Liu Y, Zhou Y, Guo AC, Cruz JA (2012). METAGENassist: a comprehensive web server for comparative metagenomics. Nucleic Acids Res.

[8] Freilich MA, Wieters E, Broitman BR, Marquet PA, Navarrete SA (2018). Species co-occurrence networks: can they reveal trophic and non-trophic interactions in ecological communities?. Ecology.

[9] Mukhopadhya I, Hansen R, El-Omar EM, Hold GL (2012). IBD: what role do Proteobacteria play?. Nat Rev Gastroenterol Hepatol.

[10] DiGiulio DB, Callahan BJ, McMurdie PJ, Costello EK, Lyell DJ, Robaczewska A (2015). Temporal and spatial variation of the human microbiota during pregnancy. Proc Natl Acad Sci USA.

[11] Bisanz JE, Enos MK, PrayGod G, Seney S, Macklaim JM, Chilton S (2015). Microbiota at multiple body sites during pregnancy in a rural Tanzanian population and effects of moringa-supplemented probiotic yogurt. Appl Environ Microbiol.

[12] Ryan MP, Pembroke JT (2018). *Brevundimonas* spp: emerging global opportunistic pathogens. Virulence.

[13] Le Chatelier E, Nielsen T, Qin J, Prifti E, Hildebrand F, Falony G (2013). Richness of human gut microbiome correlates with metabolic markers. Nature.

[14] Singh S, Karagas MR, Mueller NT (2017). Charting the maternal and infant microbiome: what is the role of diabetes and obesity in pregnancy?. Curr Diab Rep.

[15] Mullins TP, Tomsett KI, Gallo LA, Callaway LK, McIntyre HD, Nitert MD (2021). Maternal gut microbiota displays minor changes in overweight and obese women with GDM. Nutr Metab Cardiovasc Dis.

[16] Wei J, Qing Y, Zhou H, Liu J, Qi C, Gao J (2021). 16S rRNA gene amplicon sequencing of gut microbiota in gestational diabetes mellitus and their correlation with disease risk factors. J Endocrinol Invest.

[17] Zhou L, Xiao X (2018). The role of gut microbiota in the effects of maternal obesity during pregnancy on offspring metabolism. Biosci Rep.

[18] Gomez-Arango LF, Barrett HL, McIntyre HD, Callaway LK, Morrison M, Dekker Nitert M (2016). Connections between the gut microbiome and metabolic hormones in early pregnancy in overweight and obese women. Diabetes.

[19] Zhou K (2017). Strategies to promote abundance of *Akkermansia muciniphila*, an emerging probiotics in the gut: evidence from dietary intervention studies. J Funct Foods.

[20] Zhou Q, Zhang Y, Wang X, Yang R, Zhu X, Zhang Y (2020). Gut bacteria *Akkermansia* is associated with reduced risk of obesity: evidence from the American Gut Project. Nutr Metab.

[21] Xu Y, Wang N, Tan HY, Li S, Zhang C, Feng Y (2020). Function of *Akkermansia muciniphila* in obesity: interactions with lipid metabolism, immune response and gut systems. Front Microbiol.

[22] Ndongo S, Tall ML, Ngom II, Delerce J, Levasseur A, Raoult D (2019). *Olsenella timonensis* sp. nov., a new bacteria species isolated from the human gut microbiota. New Microbes New Infect.

[23] Liu B, Kleinsteuber S, Centler F, Harms H, Sträuber H (2020). Competition between butyrate fermenters and chain-elongating bacteria limits the efficiency of medium-chain carboxylate production. Front Microbiol.

[24] Coppola S, Avagliano C, Calignano A, Berni Canani R (2021). The protective role of butyrate against obesity and obesity-related diseases. Molecules.

[25] Parada Venegas D, de la Fuente MK, Landskron G, González MJ, Quera R, Dijkstra G (2019). Short chain fatty acids (SCFAs)-mediated gut epithelial and immune regulation and its relevance for inflammatory bowel diseases. Front Immunol.

[26] Tims S, Derom C, Jonkers DM, Vlietinck R, Saris WH, Kleerebezem M (2013). Microbiota conservation and BMI signatures in adult monozygotic twins. ISME J.

[27] Garcia-Mantrana I, Selma-Royo M, Alcantara C, Collado MC (2018). Shifts on gut microbiota associated to Mediterranean diet adherence and specific dietary intakes on general adult population. Front Microbiol.

[28] Escobar JS, Klotz B, Valdes BE, Agudelo GM (2014). The gut microbiota of Colombians differs from that of Americans, Europeans and Asians. BMC Microbiol.

[29] Haro C, Montes-Borrego M, Rangel-Zúñiga OA, Alcalá-Díaz JF, Gómez-Delgado F, Pérez-Martínez P (2016). Two healthy diets modulate gut microbial community improving insulin sensitivity in a human obese population. J Clin Endocrinol.

[30] Gonzalez-Perez G, Hicks AL, Tekieli TM, Radens CM, Williams BL, Lamouse-Smith ES (2016). Maternal antibiotic treatment impacts development of the neonatal intestinal microbiome and antiviral immunity. J Immunol.

[31] Khan I, Azhar EI, Abbas AT, Kumosani T, Barbour EK, Raoult D (2016). Metagenomic analysis of antibiotic-induced changes in gut microbiota in a pregnant rat model. Front Pharmacol.

